# Non-invasive continuous cardiac output monitoring in infants with hypoxic ischaemic encephalopathy

**DOI:** 10.1038/s41372-022-01495-2

**Published:** 2022-09-02

**Authors:** Aisling A. Garvey, Roisin O’Neill, Vicki Livingstone, Andreea M. Pavel, Daragh Finn, Geraldine B. Boylan, Deirdre M. Murray, Eugene M. Dempsey

**Affiliations:** 1 Department of Neonatology, Cork University Maternity Hospital, Wilton, Cork, Ireland; 2 INFANT Research Centre, University College Cork, Cork, Ireland; 3 Department of Paediatrics & Child Health, University College Cork, Cork, Ireland

**Keywords:** Paediatric neurological disorders, Translational research

## Abstract

**Objective:**

To describe early, continuous, non-invasive measures of cardiac output (CO) and evolution over time in infants with hypoxic-ischaemic encephalopathy (HIE).

**Study design:**

Prospective observational study of 44 infants with HIE (23 mild, 17 moderate, 4 severe) and 17 term controls. Infants with HIE had non-invasive CO monitoring (NICOM) continuously in the neonatal unit. Term controls had NICOM recorded at 6 and 24 h. A mixed-modelling approach was used to assess change in CO over time by group.

**Results:**

Infants with moderate HIE have significantly lower CO than the mild group at all timepoints (10.7 mls/kg/min lower, 95% CI:1.0,20.4, *p* = 0.03) which increases over time, driven by a gradual increase in stroke volume (SV). CO increased further during rewarming predominantly due to an increase in HR.

**Conclusion:**

TH has a significant impact on HR but SV appears largely unaffected. NICOM may provide a non-invasive, continuous, low-cost alternative to monitoring CO in infants with HIE however further research is warranted.

## Introduction

Hypoxic ischaemic encephalopathy (HIE) is a multi-organ pathology. While the majority of research has focused on brain injury and optimising neuroprotection, the impact of HIE and therapeutic hypothermia (TH) on the cardiovascular system is less well studied. Ischaemic changes and impaired ventricular function have been described following HIE and impaired ventricular function has been shown to correlate with the grade of encephalopathy and outcome [1–4]. Reduced cardiac output (CO) may further compound underlying brain injury due to reduced cerebral perfusion [5]. Therefore, it is important that relevant and continuous haemodynamic information is available to the clinician.

Current methods of determining cardiovascular function include blood pressure (BP) monitoring and point-of-care neonatal echocardiograms. BP has long been used as a surrogate for cardiovascular function however changes in CO may occur despite a stable BP and BP is a poor surrogate marker of blood flow [6–8]. Echocardiography has become the primary method of measuring CO in infants in the neonatal unit. Echocardiography provides a snapshot of haemodynamic function at a specific time point. Repeated clinical examination and monitoring urine output also provide useful information regarding CO however ideally, monitoring should be non-invasive, accurate, reliable, and, importantly, continuous. Non-invasive methods of continuously monitoring CO are routinely used in adult intensive care units, and are now being evaluated in neonatal intensive care units [8–10]. Two different methods of measuring thoracic impedance and thus CO are currently available: bioimpedance, of which electrical cardiometry (EC) is the most commonly used technique, and bioreactance (BR) [11]. BR is a technology which measures phase shifts in the aorta to detect changes in flow between systole and diastole. From this, stroke volume (SV) can be obtained and, in combination with heart rate (HR), CO can be calculated. BR technology has been validated in the adult population and values correlate with invasive measures of CO [12–15]. It is less influenced by artefact and electrical noise than other non-invasive measures of CO and is unaffected by ventilation status [16, 17]. Its use has been assessed in infants and young children undergoing cardiac surgery [18]. In the neonatal population, it has been shown to correlate with echocardiogram-derived measures of left ventricular output [19, 20]. Its use in HIE has been limited to assessing the effect of TH on CO [8].

Three studies have used non-invasive monitoring to assess the effect of therapeutic hypothermia on CO in infants with moderate and severe HIE-one using BR and two studies using EC [8–10]. No study has examined the ability of thoracic impedence to measure CO in infants with mild HIE or to describe the changes in CO over time in infants with all grades of HIE. Thus, the aim of this study was to describe early CO values in infants with all grades of HIE and to assess if its evolution over time differs between grades of HIE and compared with healthy term infants.

## Methods

This was part of a larger prospective observational study conducted in Cork University Maternity Hospital, Ireland between November 2017 and March 2020 in which infants with all grades of HIE and healthy term controls had multi-modal monitoring during their stay in the neonatal unit to include electroencephalogram (EEG), near infrared spectroscopy (NIRS), non-invasive cardiac output monitoring (NICOM), echocardiography, magnetic resonance imaging (MRI) and blood biomarkers. This study aimed to examine the evolution of non-invasive physiological biomarkers over time in infants with HIE and determine whether these markers, individually or in combination, are useful in the prediction of outcome. EEG data from this study has been published previously but NICOM data has not been published [21].

NICOM was obtained using the Cheetah Starling NICOM ™ device (Cheetah Medical, Vancouver, Washington, USA) which uses BR technology. Four sensors are applied to the infant’s thorax (left and right, upper and lower) in a manner that “boxes” the heart. The NICOM device averages measurements of CO, HR, and SV every 60 s. Following the recording period, data was exported to Microsoft Excel. Mean CO and SV were divided by the infant’s birth weight to provide measurements in mls/kg/min (CO) and mls/kg (SV). HR was reported as beats/minute.

All infants had an echocardiogram performed during NICOM monitoring using the GE Vivid-I Portable Ultrasound (GE Healthcare, Chicago, Illinois, USA). Echocardiograms were analysed off-line by a neonatologist with echocardiogram experience (ED) blinded to the study group. SV was calculated using the standard equation below:

$$\pi \times \left. {\left( {\frac{{VOT}}{2}} \right.} \right)^2 \times VTI$$

*VOT, ventricular outflow tract; VTI, velocity time ingegral*

CO in mls/kg/minute was then calculated as follows:

$$\frac{{HR \times SV}}{{birthweight}}$$

### Infants with HIE

Infants >36 weeks gestation at birth were included if they had evidence of HIE to include one or more of: an Apgar score of <5 at 5 min of life, requirement of continued resuscitation at 10 min of life or pH of <7.1/base deficit of >16/lactate of >9 mmol/L on cord or first post-natal blood gas plus clinically evolving encephalopathy defined as the presence of abnormal neurological findings using the modified Sarnat score at 1 h of life.

TH was commenced before 6 h of life in all infants with moderate and severe HIE. Infants with mild HIE did not receive TH. It is practice in our unit that infants undergoing TH receive low dose morphine infusion (10–20 mcg/kg/h) which is titrated according to clinical response. Infants with mild HIE were initially commenced on IV fluids at a rate of 60 mls/kg/day until lactate normalised and feeds were established. Infants with moderate and severe HIE were commenced on IV fluids at 40–60 mls/kg/day and titrated to electrolytes and urine output.

Following parental consent, NICOM monitoring was commenced as soon as possible after birth and continued for approximately 24–36 h in infants with mild HIE, and throughout TH and the rewarming phase in infants with moderate and severe HIE. 1 h data epochs were extracted for analysis at 6, 9, 12, 18, 24, 36, 48, 72 and 84 h of age for each infant where available.

Infants with HIE had an echocardiographic assessment of CO performed in the first 48 h of life and if NICOM monitoring continued beyond this time they had a second echocardiogram after 48 h of age.

### Healthy term infants

Healthy term infants were recruited on the post-natal ward (PNW). Infants >36 weeks gestation were eligible for recruitment if they did not require resuscitation at birth, had normal cord pH values and had an apgar score of >8 at 5 min of life. These infants had NICOM recorded on the PNW at 6 and 24 h of life for 1–2 h duration.

Control infants had an echocardiogram performed on the PNW during one of the monitoring time points.

### Statistical analysis

Statistical analysis was performed using IBM SPSS Statistics (version 26.0, IBM Corp, Armonk, NY, U.S.A.) and Stata (version 15.1, StataCorp LP, TX, U.S.A.). Continuous variables were described using mean and standard deviation (SD) or median and interquartile range (IQR) and categorical variables using frequency and percentage.

To investigate and compare how CO, HR, and SV changed over time (from 6 to 48 h after birth) for the mild and moderate HIE groups, a mixed modelling approach was used. Due to limited data in the severe group, they were excluded from the analysis. The optional functional form of the trajectory over time was identified from the family of polynomial functions (a straight line, a quadratic curve, and a cubic curve). A bottom-up strategy was used, starting with an empty random intercepts model and then adding each fixed effect (linear, quadratic, cubic) followed by its corresponding random time effect (linear, quadratic, cubic), in turn. Model fit was evaluated using the deviance statistic (−2 log-likelihood) and the Akaike Information Criterion. To investigate if changes over time differed by HIE group, the fixed effects of HIE group and the interactions of HIE group by time (linear, quadratic, and cubic, as appropriate) were added to the mixed model in a sequential manner. Time was treated as a continuous variable in this analysis.

To investigate if changes in the NICOM measures (CO, HR, and SV) between 6 and 24 h differed between the mild HIE and control groups, random effects modelling was used. Each model included time, group, and time*group as fixed effects and infant as a random effect. Time was treated as a categorical variable in this analysis.

One-way Analysis of Variance (ANOVA) was used to compare cardiac output, heart rate and stroke volume at 24 h between the control, mild HIE and moderate HIE groups. If statistically significant differences were found, post-hoc pairwise comparisons were performed using Tukey’s Honestly Significant Difference test.

All tests were two-sided and *p*-values <0.05 were considered statistically significant.

Bland-Altman analysis was used to compare echocardiogram and NICOM measures of CO. As the data included repeated measures, bias and limits of agreement (LoA) were calculated using random effects modelling [22, 23]. The model included a constant term (no fixed effects) and a random effect for infants. The dependent variable was the difference between the paired echocardiographic and NICOM CO measurements. The intercept from the fitted model represented the mean bias and the standard deviation of the bias (SD) was calculated as the square root of the sum of the between- and within-infant variances. Using these, the random effects limits of agreement were calculated using the conventional Bland-Altman limits of agreement formula (bias ± 1.96*SD). Limits of agreement provide an estimation of how precise the measurements are i.e the repeatability and reproducibility. Percentage bias was calculated as (bias/mean value of the two methods) x 100. Percentage bias examines the mean difference between the measurements and gives an estimation of the accuracy between the methods. The percentage error refers to (1.96 x  SD/mean value of the two methods) x 100 and examines the precision of NICOM and whether the two measurement techniques are interchangeable. A percentage error of 30% is clinically acceptable for interchangeability between the methods [24].

## Results

Sixty-one infants were included in this study (17 controls, 23 mild HIE, 17 moderate HIE, 4 severe HIE). Demographic data is outlined in Table 1.

**Table 1:** Demographic information of included infants.

	Controls *n* = 17	Mild *n* = 23	Moderate *n* = 17	Severe *n* = 4
GA, *weeks*	39.4 (37.6–40.4)	40.4 (38.3–40.9)	38.6 (37.6–40.0)	39.6 (38.4–40.6)
BW, *kg*	3.7 (3.4–3.8)	3.3 (3.0–3.7)	3.1 (2.8–3.6)	3.7 (3.4–3.9)
Sex: Male	6 (35)	15 (65)	9 (53)	2 (50)
ROM, *hours*	2.4 (1.2–12.2)	14.0 (3.5–27.6)	7.0 (4.1–16.7)	13.0 (4.0-13.0)
Onset of labour				
Spontaneous	7 (41)	14 (61)	8 (47)	4 (100)
Induction	8 (47)	7 (30)	7 (41)	
Pre-labour LSCS	2 (12)	2 (9)	2 (12)	
Mode of Delivery				
SVD	8 (47)	5 (22)	4 (24)	2 (50)
Instrumental	5 (29)	10 (43)	6 (35)	
EmLSCS	2 (12)	6 (26)	5 (29)	2 (50)
Pre-labour LSCS	2 (12)	2 (9)	2 (12)	
1 minute Apgar				
0–4	0	11 (48)	17 (100)	4 (100)
5–7	1 (6)	8 (35)		
8–10	16 (94)	4 (17)		
5 minute Apgar				
0–4		2 (9)	9 (53)	4 (100)
5–7		7 (30)	8 (47)	
8–10	17 (100)	14 (61)		
Biochemistry^a^				
Lowest pH		7.0 (6.9–7.1)	6.9 (6.8–7.1)	7.1 (6.9-7.1)
1st Postnatal Lactate		10.7 (5.4–13.3)	10.9 (7.6–13.7)	14.9 (9.1–17.6)
24 h Lactate		2.4 (1.9–3.4)	1.7 (1.3–3.4)	2.5 (1.3–2.5)
48 h Lactate		2.1 (1.8–2.1)	1.4 (1.1–2.1)	1.1
Medications				
AEDs	0	0	3 (18)	2 (50)
Inotropes	0	0	1 (6)	2 (50)

^a^Biochemistry not recorded for control group.

*GA* gestational age, *BW* birthweight, *ROM* rupture of membranes, *LSCS* lower section caesarean section, *SVD* spontaneous vaginal delivery, *EmLSCS* emergency LSCS, *AEDs* anti-epileptic drugs. Categorical variables are represented as frequencies and percentage. Continuous variables are represented as median and interquartile range.

### Summary measures of CO, HR and SV over time

**Fig. 1:**
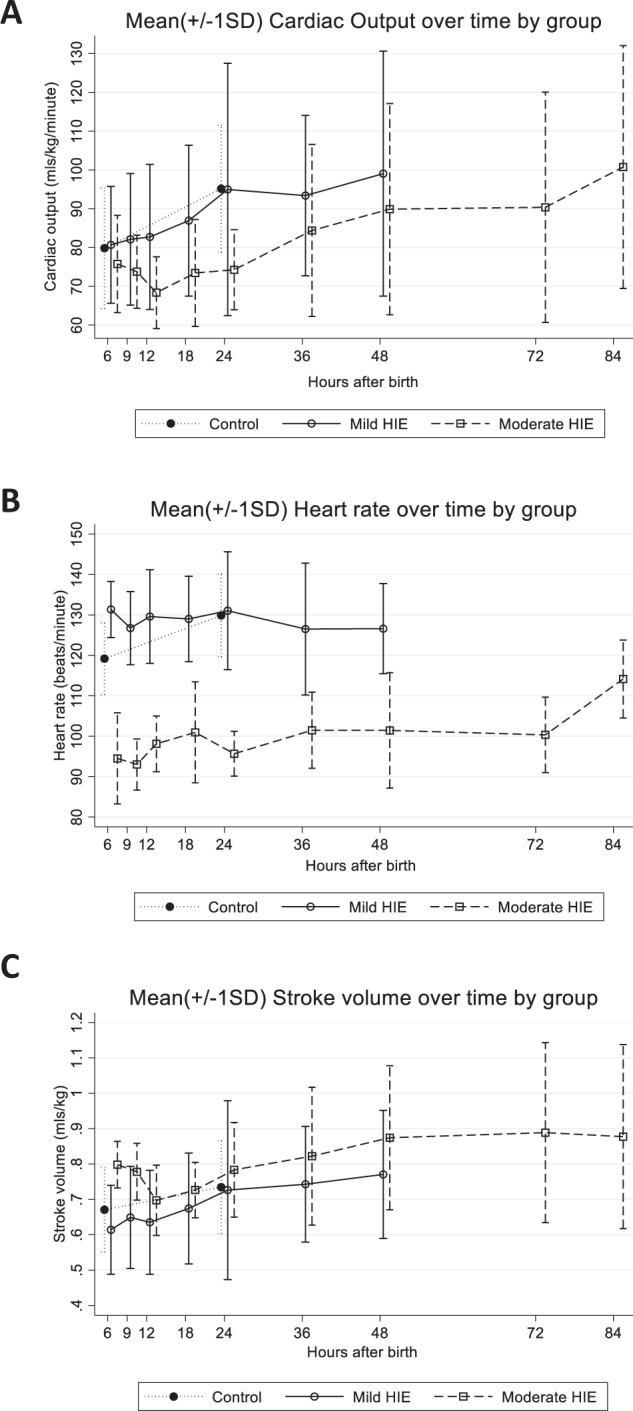
Cardiac Output, Heart Rate and Stroke Volume over time by group. Mean and Standard Deviation values of available Cardiac Output (**A**), Heart Rate (**B**) and Stroke Volume (**C**) at each timepoint by group (controls *n* = 17, mild HIE *n* = 23, moderate HIE *n* = 17). Summary measures of CO, HR, and SV and number of included infants at each timepoint in each group are included in Supplementary Material 1.

Summary measures of CO (mls/kg/min), HR (beats/min), and SV (mls/kg) over time in all infant groups are outlined in Supplementary Material S1. Figure 1 depicts changes in mean CO (mls/kg/min), HR (beats/min), and SV (mls/min) over time in control infants and mild and moderate HIE groups. 4 infants were included in the severe group. Early recordings were not possible in this group due to the challenges in obtaining early informed parental consent. Due to the limited data available in this group, they have been included in the descriptive analysis of the results below but omitted from further statistical analysis. Infants with mild HIE and controls have similar CO values over the first 24 h. Infants with moderate and severe HIE have lower CO values which increase gradually throughout TH and again during rewarming. Although SV increases in both groups, during the rewarming phase, this increase is predominantly due to an increase in HR.

### Evolution of CO, HR and SV over time

**Fig. 2:**
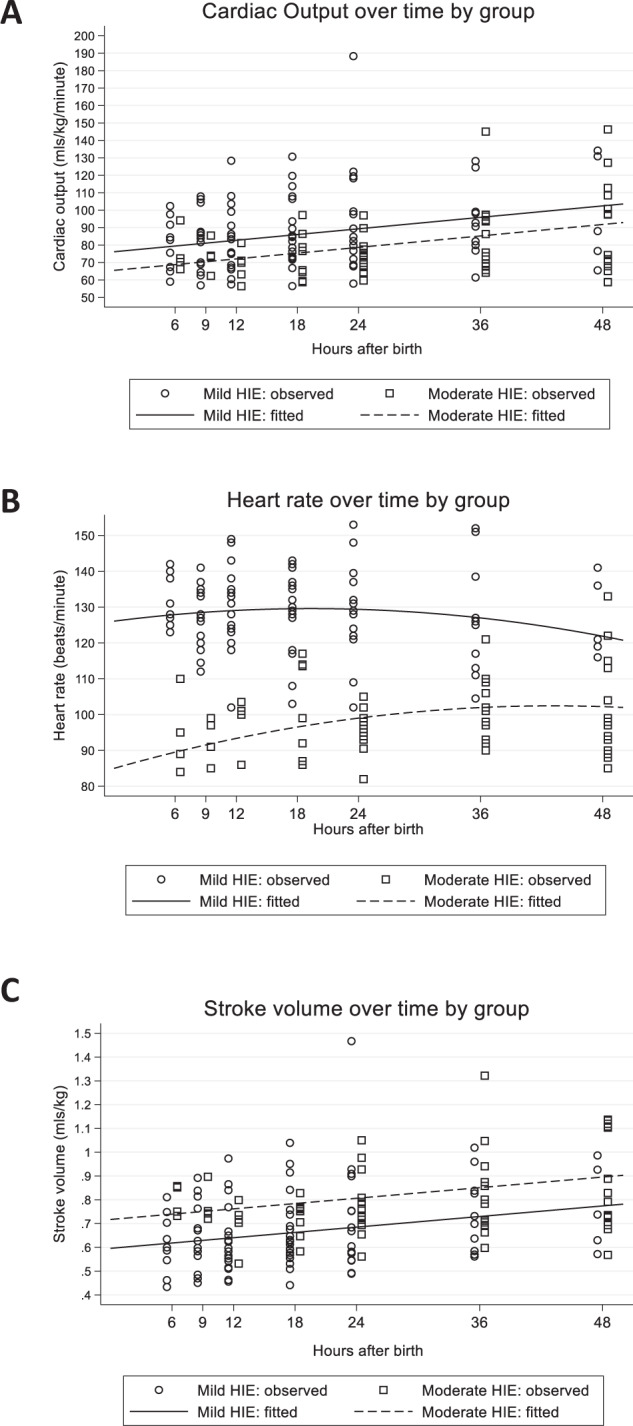
Change in Cardiac Output, Heart Rate, and Stroke Volume over time in infants with mild HIE and with moderate HIE (during therapeutic hypothermia). Mixed-modeling approach to investigate change in CO (**A**), HR (**B**), and SV (**C**) over time. CO, HR, and SV increased in both groups over time. CO was higher in the mild group at all timepoints (*p* = 0.031). SV was higher in the moderate group at all timepoints (*p* = 0.005).

For cardiac output, the best fitting model included a fixed and random intercept, a fixed and random linear time effect and a fixed group effect. Changes over time did not differ between the two groups (*p* = 0.683 for group*time interaction).(Fig. 2A). The fitted lines were CO = 79.5 + 0.5*(hours-6) for the mild group and CO = 68.8 + 0.5*(hours-6) for the moderate group. CO increased over time in both the mild and moderate groups by 0.5 mls/kg/min (95% CI:0.2, 0.9) for every hour increase in age (*p* = 0.001). Across all timepoints, CO was on average 10.7 mls/kg/min (95% CI:1.0, 20.4) higher in the mild HIE group (*p* = 0.031).

For heart rate, the best fitting model was quadratic with fixed and random effects for the intercept, linear time, and quadratic time, a fixed group effect, and a fixed group*time effect. Mean heart rate increased in both groups over time with the rate of increase slowing down over time (decelerating positive curves). (Fig. 2B). The fitted curves were 127.915 + 0.250*(hours-6)−0.009 (hours-6)^2^ for the mild group and 89.525 + 0.696*(hours-6)−0.009 (hours-6)^2^ for the moderate group. The instantaneous linear rate of change at 6 h after birth was significantly higher in the moderate group (0.696 vs 0.250 in the mild group, *p* = 0.001). The rate of deceleration in both groups was not significantly different (*p* > 0.05 for group*(time-6)^2^ interaction and hence not included in final model).

For stroke volume, the best fitting model was linear with a fixed and random intercept, a fixed and random linear time effect, and a fixed group effect. Changes over time did not differ between the two groups (*p* = 0.249 for group*time interaction).(Fig. 2C). The fitted lines were SV = 0.618 + 0.004*(hours-6) for the mild group and SV = 0.739 + 0.004*(hours-6) for the moderate group. SV increased over time in both the mild and moderate groups by 0.004 mls/kg (95% CI:0.001, 0.006) for every hour increase in age (*p* = 0.002). Across all timepoints, SV was on average 0.121 mls/kg (95% CI:0.037, 0.205) higher in the moderate HIE group (*p* = 0.005).

### Mild HIE and controls at 6 and 24 hours

**Fig. 3:**
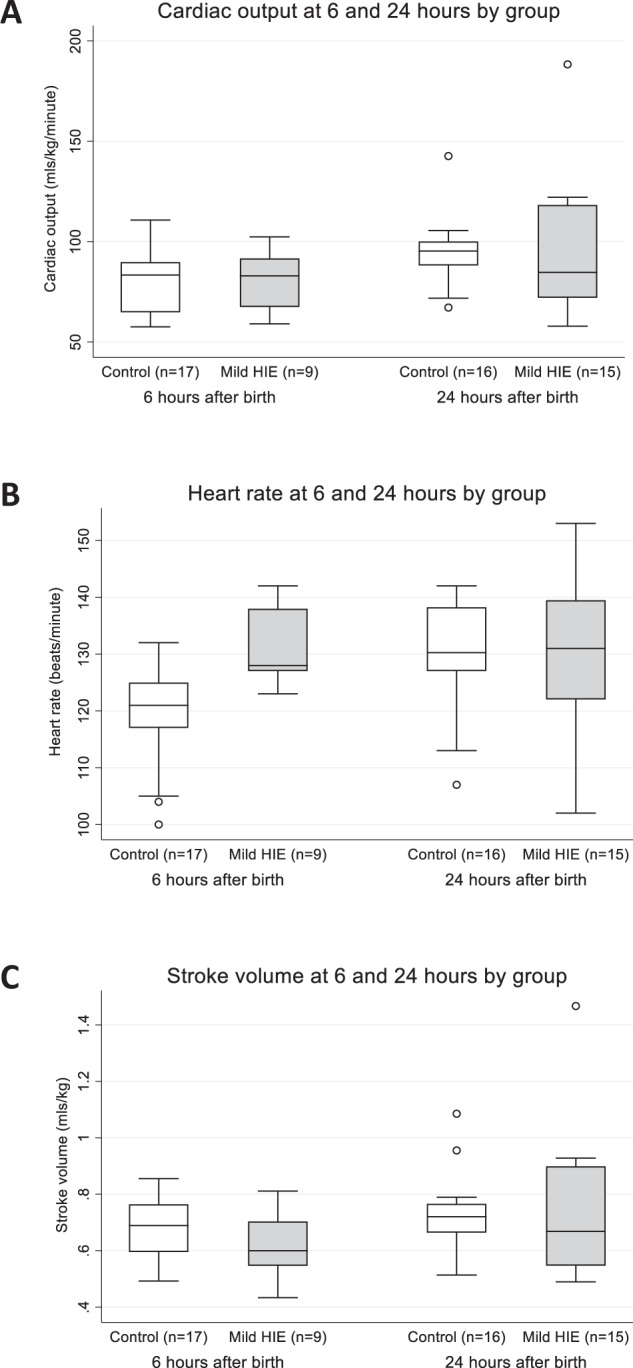
Evolution of Cardiac Output, Heart Rate and Stroke Volume over time in Control and Mild HIE groups. Evolution of CO is depicted in (**A**), HR in (**B**) and SV in (**C**). Random effects modelling to investigate changes in NICOM measures over time. Increase over time was seen in CO (*p* = 0.001) and SV (*p* = 0.018), this increase did not differ between groups. At 6 h, mild HIE had a higher HR (*p* = 0.034). There was no difference at 24 h (*p* = 0.703). Boxes represent median (IQR) with whiskers representing 1.5 × IQR. Circles represent outliers.

To examine this trend further, we assessed the cardiovascular changes in infants with mild HIE and controls at 6 and 24 h of life. Changes in cardiac output over time did not differ between groups (*p* = 0.947 for time*group). Cardiac output increased in both groups from 6 to 24 h of life (p = 0.001 for time) with CO on average 15.7 mls/kg/min (95% CI: 6.4, 25.0) higher at 24 h (Fig. 3A).

Changes in heart rate over time were different for the mild HIE and control groups (*p* = 0.046 for time*group). In the control group, HR increased significantly from 6 to 24 h (regression coefficient (95% CI): 11.0 (7.0, 15.0), *p* < 0.001). There was no significant change over time in the mild HIE group (regression coefficient (95% CI): 3.5(−2.8, 9.7), *p* = 0.274).

At 6 h, infants with mild HIE had a significantly higher HR compared to the controls (regression coefficient (95% CI): 9.0(0.7, 17.4), *p* = 0.034)(Fig. 3B). There was not a significant difference between the two groups at 24 h (*p* = 0.703).

Changes in SV over time did not differ between groups (*p* = 0.427 for time*group). In both groups, SV increased between 6 and 24 h (p = 0.018) with SV on average 0.08 ml/kg (95% CI: 0.01, 0.15) higher at 24 h (Fig. 3(C)).

### Controls, mild HIE and moderate HIE at 24 hours

**Fig. 4:**
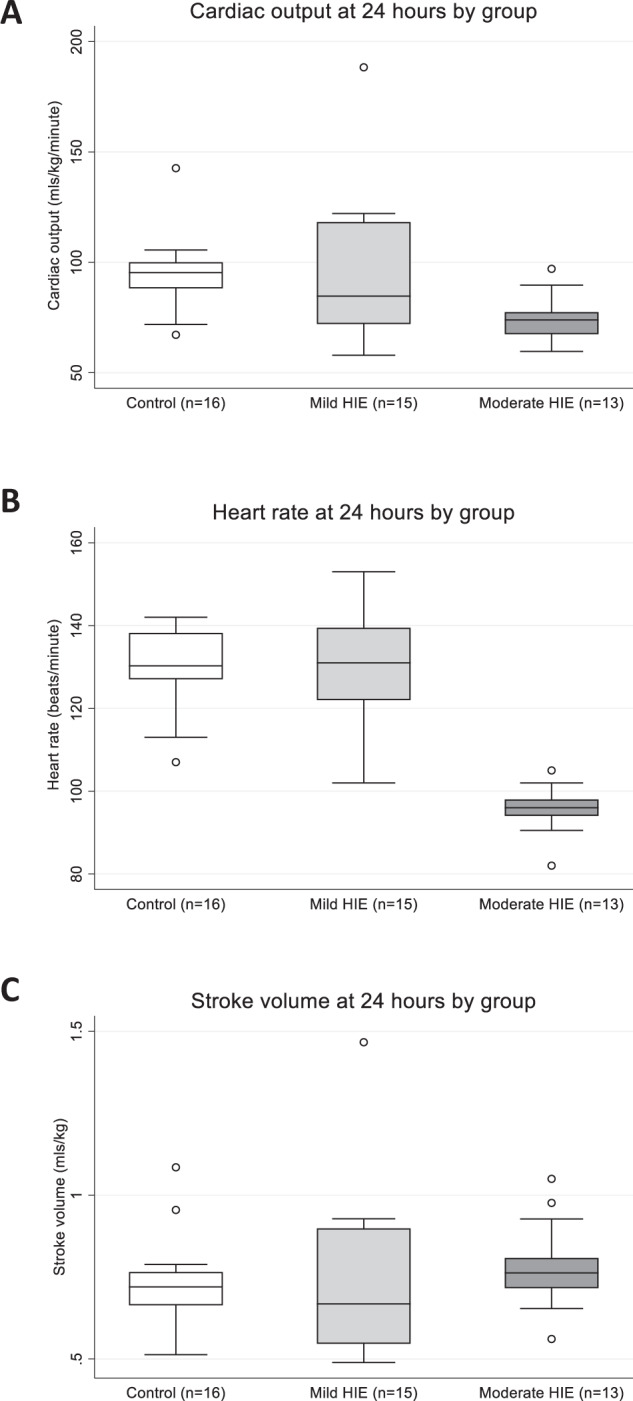
Cardiac Output, Heart Rate and Stroke Volume at 24 h of life in control, mild HIE and moderate groups. CO for each group is depicted in (**A**), HR in (**B**) and SV in (**C**). CO and HR were lower in the moderate group compared with the mild group (*p* = 0.046, *p* < 0.001)) and control group (*p* = 0.040, *p* < 0.001). No differences in SV were found (*p* = 0.677). *p*-values derived from one-way ANOVA and Tukey’s Test. Boxes represent median (IQR) with whiskers representing 1.5 × IQR. Circles represent outliers.

Boxplots are presented in Fig. 4 for cardiac output, heart rate, and stroke volume at 24 h for the control, mild HIE, and moderate HIE groups.

Cardiac output at 24 h was significantly different between the groups (*p* = 0.025). Pairwise comparisons showed that CO was significantly lower in the moderate HIE group compared to both the mild HIE (adjusted *p* = 0.046) and control group (adjusted *p* = 0.040). No difference was found between the mild HIE and control group (adjusted *p* > 0.999).

Heart rate at 24 h was also significantly different between the groups (*p* < 0.001). The differences were between the moderate HIE group and both the mild HIE (adjusted *p* < 0.001) and control group (adjusted *p* < 0.001) with heart rate being significantly lower in the moderate HIE group. No difference was found between infants with mild HIE and controls (adjusted *p* = 0.953).

No differences in SV at 24 h between groups were found (*p* = 0.677).

### Agreement of echocardiogram and NICOM measures of CO

Sixty four echocardiogram-NICOM paired comparisons across 54 infants were included in the analysis (16 controls, 22 mild, 13 moderate, and 3 severe). The Bland-Altman plot is presented in Supplementary Material S2. The bias and limits of agreement were 21.7 mls/kg/min (95% limits of agreement −36.6 to 80.0 mls/kg/min). The percentage bias was 21.2% and the percentage error was 56.8%.

## Discussion

This study describes the evolution of CO using NICOM over time in newborns with all grades of HIE compared with healthy term controls. CO was similar in infants with mild HIE and controls, although infants with mild HIE had a significantly higher HR at 6 h of life. Infants with moderate HIE had a significantly lower CO than the mild group at each timepoint whilst CO in both groups increased over time. The lower CO identified initially in the moderate group increased gradually over the TH period, mainly due to an increase in SV and a further increase in CO during the rewarming period predominantly due to an increase in HR. These findings suggest an effect of both the underlying encephalopathy and subsequent treatment with TH, on cardiovascular haemodynamics.

In addition to its neurological implications, HIE may also result in significant haemodynamic alterations. The effect of HIE on cardiac function is 2-fold. Firstly, injury to the myocardium may result in ventricular dysfunction which may lead to reduced CO and pulmonary venous hypertension [5]. Secondly, pulmonary arterial hypertension may result from increased pulmonary vascular resistance secondary to hypoxia-induced vasoconstriction of the pulmonary vessels [25]. As cerebral autoregulation may also be impaired in infants with HIE, increased recognition of cardiovascular impairments and the subsequent effects on cerebral perfusion and thus outcome is important [26].

Infants with mild HIE had a significantly higher HR than controls at 6 h. By 24 h, CO, HR and SV were similar in both the mild and control groups. The normal physiological trend is that CO increases over the first 48 h, predominantly due to an increase in SV, and this increase co-incides with increases in functional measurements of the right and left ventricles [27, 28]. HR is elevated at time of birth but reduces significantly in the first 15–30 min of life and gradually increases over the first week [29]. This is in keeping with our finding of CO, SV, and HR increasing from 6 to 24 h in the control group. The increased HR at 6 h in infants with mild HIE may be explained by over-activation of the sympathetic nervous system, consistent with previous findings [30, 31]. Although not significant, the slightly reduced SV may be due to reduced preload as a consequence of the increased HR or increased afterload due to an effect of acute asphyxia on pulmonary and systemic vascular resistance [32]. However, we can only postulate as detailed functional echocardiography was not performed at this early stage to allow us to determine this.

Infants with moderate HIE have a significantly lower CO over time when compared to infants with mild HIE. However, CO increases steadily over time in the moderate group receiving TH, with a greater rise noted during rewarming due to increasing heart rate primarily. We found that SV increases gradually in infants with moderate and severe HIE during TH, suggesting that the reduced SV seen was not due to TH alone. TH aims to reduce cerebral metabolic demand and thus reduce injury caused by secondary reperfusion. Bradycardia is a known side-effect [33]. In our cohort, HR remained stable throughout the cooling phase but increased during rewarming. Our group has previously demonstrated significant differences in measures of heart-rate variability (HRV) between infants with moderate HIE in the pre and post TH era suggesting increased activity of the parasympathetic nervous system which may also explain the reduced HR seen in our cohort during TH [31]. It is also important to consider the effect of medications such as morphine when interpreting our findings. Bradycardia is a known side-effect of morphine [34] and all infants with moderate and severe HIE received morphine during TH as per our unit’s protocol, albeit at different doses and titrated according to clinical response.

Infants with moderate and severe HIE had a significant increase in HR and CO during the rewarming phase. This finding is in keeping with previously published studies. Forman et al. showed that NICOM measures of CO remained stable during TH in 20 infants with HIE but CO and HR significantly increased during rewarming [8]. Similarly, Wu et al. used EC to assess CO prior to and after rewarming in 20 infants with moderate and severe HIE and found that CO increased significantly during rewarming driven by a significant increase in HR [9].

Eriksen et al. used EC to assess the effect of early TH on CO in a group of 15 infants with moderate and severe HIE compared with 10 healthy term controls [10]. Five infants with HIE had haemodynamic measurements recorded prior to initiation of TH. Compared with the control group, these infants had a lower CO and SV suggesting an effect of the original injury. CO reduced further once infants were cooled and this was largely due to a reduction in HR. All infants undergoing TH in our study had TH commenced prior to initiating NICOM monitoring so we cannot extrapolate whether the reduced CO was solely due to the effects of TH or whether it was compounded by the degree of encephalopathy. Activation of the parasympathetic nervous system is a hallmark of a moderate grade of encephalopathy evident by bradycardia [30]. In addition, perinatal asphyxia itself results in myocardial dysfunction [2, 32, 35]. Reduction in CO along with the increased pulmonary and systemic vascular resistance associated with acute asphyxia [32] may be a protective mechanism by which the risk of cerebral hyperperfusion is reduced. However, maintenance of an appropriate CO is required to maintain adequate cerebral perfusion and oxygen delivery to avoid further brain injury [4, 5]. This delicate balance is crucial in optimising neuroprotection and so a continuous monitoring tool such as NICOM may be an important management tool [8, 36, 37]. Understanding the complex interaction between CO and cerebral blood flow in the future may allow us to individualise management of these infants.

NICOM has the potential to provide this information and has gained much interest in the neonatal population due to its ease of application and provision of a continuous insight into cardiac output. It is less affected by artefact than other non-invasive measures of CO and is not affected by ventilatory status [12–16, 38]. NICOM readings are not affected by reduced body temperature and this finding is also supported by previous studies in animals [8, 39].

NICOM use in neonates remains predominately in a research setting due to difficulties in validating its use against gold standard measurements of CO which are invasive and challenging. Echocardiography remains the most accepted method of measuring cardiovascular function in infants in the neonatal unit. When compared with echocardiogram-CO, NICOM consistently under reads echocardiography measurements by approximately 27–30% [8, 19, 20]. Our percentage bias of 21.2% is less than previously reported however, in keeping with previous studies, the percentage error of 56.8% exceeded the clinically acceptable percentage error of 30% implying that echocardiogram-CO and NICOM-CO are not interchangeable [11]. This may be explained as follows. Firstly, the NICOM system uses an algorithm developed to measure CO in adults and extrapolates it to neonatal measurements which may account for lower NICOM measurements [39]. Another consideration is that echocardiogram-CO relies on measurements of the aortic size and velocity of blood flow which can vary depending on exact site of measurement. This may result in echocardiographic measurements of CO slightly overestimating CO [19].

Nonetheless, NICOM may have considerable advantages. Predominantly, it doesn’t require expert training to perform or interpret the measurements. Secondly, echocardiography only allows a window into cardiac function at a single snapshot in time. Cardiac function is dynamic. Cardiovascular dysfunction, particularly right ventricular dysfunction, is an independent risk factor for adverse outcome [4]. Alterations in BP, physical examination, and urine output, although important clinical signs, are late markers of impaired myocardial function. NICOM has the potential to be a useful tool in the NICU as a trend monitor allowing for early recognition of infants at risk and thus early implementation of appropriate interventions to improve outcome. Furthermore, it may be helpful in monitoring the subsequent response to treatment. Non-invasive monitoring may also have important healthcare economic considerations. Echocardiograms cost approximately $1000–$3000 dollars per examination whereas NICOM sensors are much less expensive and may therefore provide a low-cost alternative to monitoring CO [40].

This study is limited by small numbers of infants in each group and by varying durations of monitoring. Only 4 infants in our cohort had severe HIE making it difficult to interpret these findings but their trend in CO, HR, and SV are similar to infants in the moderate group. Due to challenges associated with obtaining early informed consent in this cohort, it was not possible to obtain CO measurements at all timepoints in all infants across the 4 groups. Furthermore, we do not have detailed functional echocardiographic measurements on all infants and are therefore unable to determine the presence of LV dysfunction or other underlying pathophysiological mechanisms responsible for the differences seen in NICOM measurements. However, this is the first study to describe the early evolution of cardiac output changes in infants of all grades of HIE compared to controls. Long-term follow-up is currently underway which will allow us to further examine whether these changes in CO are important in the prediction of outcome.

In conclusion, infants with mild HIE have a higher HR but similar CO to healthy infants at 6 h of life and by 24 h, there was no difference in either parameter. Infants with moderate HIE undergoing TH have a significantly lower CO and HR when compared with the mild and control groups. During TH, CO increases gradually over time driven by a gradual increase in SV (possibly reflecting improved diastolic function), and by a significant increase in HR during the rewarming phase. Cardiovascular compromise is a known complication of HIE and is an independent risk factor for adverse outcome. Improved and accessible methods of monitoring cardiovascular function are required. NICOM may provide a non-invasive, continuous, low-cost alternative to monitoring CO in infants with HIE. However further research in this area of newborn care is essential prior to its use.

## Supplementary information

Supplementary Material S1. Summary measures of CO, HR and SV over time per group

Supplementary Material S2. Bland-Altman analysis of echocardiogram and NICOM measurements of CO (mls/kg/min)

## Data Availability

For the current study, it is not possible to share the datasets. The clinical data is collected under a written proxy consent from the participants’ guardians/parents, which did not include permission for sharing or open data. To be allowed to share this data under Irish Health Research Regulations we are required to re-consent families or to obtain approval by the Health Regulation Consent Declaration Committee.
