# Supplementary figures and images for: Non-invasive continuous cardiac output monitoring in infants with hypoxic ischaemic encephalopathy
Source: J Perinatol. 2022 Sep 2;42(12):1622–9. doi: 10.1038/s41372-022-01495-2 (PMC9712087; doi:10.1038/s41372-022-01495-2)

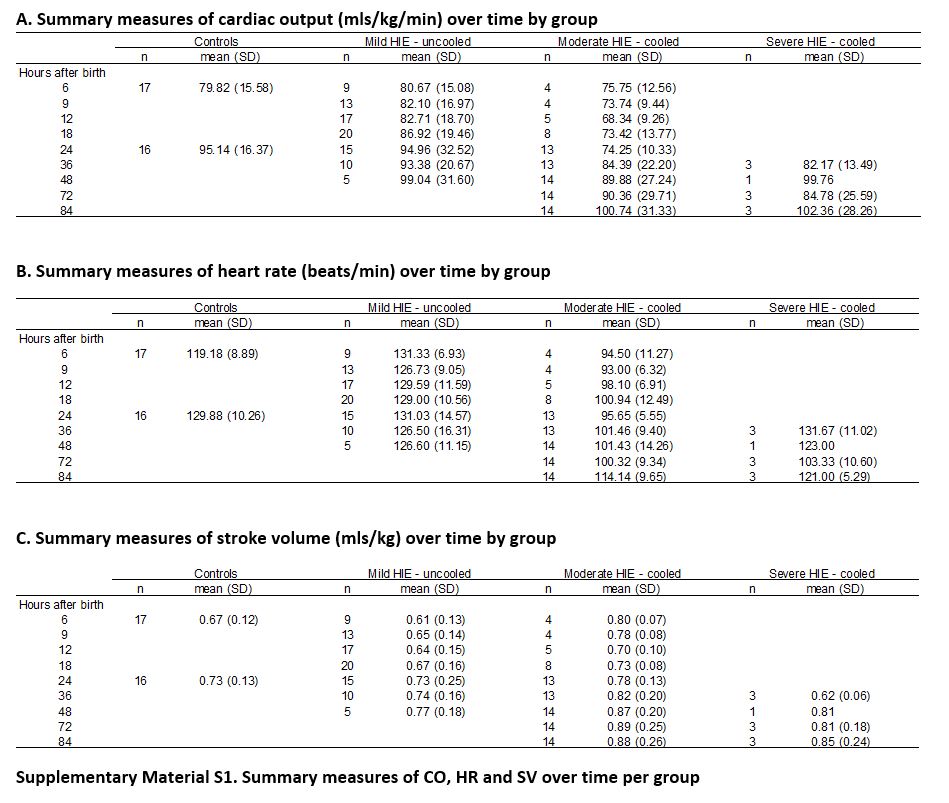

Supplement: Supplementary file 1 — Supplementary Material S1. Summary measures of CO, HR and SV over time per group [file 41372_2022_1495_MOESM1_ESM.jpg]

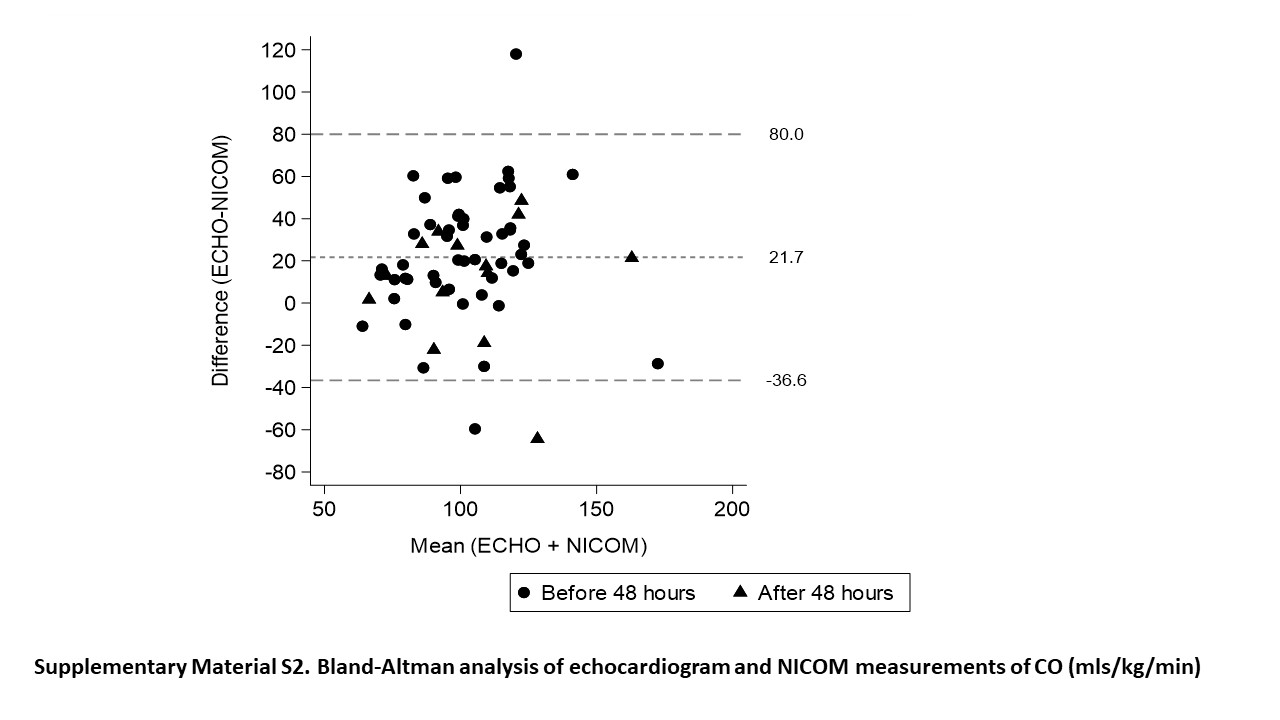

Supplement: Supplementary file 2 — Supplementary Material S2. Bland-Altman analysis of echocardiogram and NICOM measurements of CO (mls/kg/min) [file 41372_2022_1495_MOESM2_ESM.jpg]
